# Involvement of the subthalamic nucleus in engagement with behaviourally relevant stimuli

**DOI:** 10.1111/j.1460-9568.2009.06635.x

**Published:** 2009-03

**Authors:** Paul Sauleau, Alexandre Eusebio, Wesley Thevathasan, Kielan Yarrow, Alek Pogosyan, Ludvic Zrinzo, Keyoumars Ashkan, Tipu Aziz, Wim Vandenberghe, Bart Nuttin, Peter Brown

**Affiliations:** 1 Sobell Department of Motor Neuroscience and Movement Disorders, Institute of Neurology, University College LondonLondon WC1N 3BG, UK; 2 Department of Psychology, City UniversityLondon, UK; 3 Neurosurgery, King’s College HospitalLondon, UK; 4 Department of Neurosurgery, John Radcliffe HospitalOxford, UK; 5 Department of Neurology, University Hospitals LeuvenLeuven, Belgium; 6 Neurosurgery, University Hospitals LeuvenLeuven, Belgium

**Keywords:** action preparation, basal ganglia, behavioural relevance, local field potentials, subthalamic nucleus

## Abstract

In this study we investigate how the basal ganglia (BG) may process the behavioural relevance of environmental cues by recording local field potentials (LFPs) in the subthalamic nucleus of patients with Parkinson’s disease who had undergone implantation of electrodes for deep brain stimulation. Fourteen patients were recorded as they performed a paradigm dissociating warning cue presentation from programming related to execution of specific tasks. Target and non-target warning cues of differing behavioural relevance were contrasted, and we evaluated if warning cue-evoked activities varied according to whether the eventual task to be performed was motor or cognitive and whether patients were receiving or withdrawn from dopaminergic therapy. Warning cues evoked a complex temporal sequence of activities with three epochs over the 760 ms following the onset of the warning cue. In contrast to the initial evoked LFP, evoked activities over two later periods were significantly influenced by behavioural relevance and by treatment state. The early activity was likely related to the initial orientating of attention induced by a novel target, while the delayed responses in our paradigm may reflect processing related to the non-motor resource implications of cues. The results suggest that the BG are intimately involved in the evaluation of changes in the environment and of their behavioural significance. The latter process is partly modulated by dopamine. Weakness in this function might contribute to the behavioural impairment that can follow BG lesions and surgery.

## Introduction

The basal ganglia (BG) have been implicated in a variety of motor, cognitive and affective operations (Middleton & Strick, 2000; Baunez *et al.*, 2001; Saint-Cyr, 2003; Temel *et al.*, 2005; Brucke *et al.*, 2007). In humans, neurosurgical implantation of electrodes in patients with motor impairment provides an opportunity to record premovement activity directly from the BG. Local field potential (LFP) recordings from the human subthalamic nucleus (STN) demonstrate population activity that precedes internally generated and externally paced voluntary movement (Cassidy *et al.*, 2002; Priori *et al.*, 2002; Paradiso *et al.*, 2003; Kuhn *et al.*, 2004; Alegre *et al.*, 2005; Doyle *et al.*, 2005; Alonso-Frech *et al.*, 2006; Devos & Defebvre, 2006; Androulidakis *et al.*, 2007). It has hitherto been assumed that such activity reflects aspects of motor preparation and programming. Even the activity elicited by warning cues has been viewed in this light, as such cues have been informative of forthcoming movement, allowing the early preparation of action prior to an imperative cue (Williams *et al.*, 2003, 2005). This seems a reasonable interpretation of cue-evoked activity, as there is accompanying reaction time improvement suggesting some motor processing has been accomplished prior to the imperative cue (Williams *et al.*, 2005).

However, the above findings do not exclude an additional involvement of STN-evoked activity in the evaluation and management of the behavioural relevance of environmental cues and their non-motor resource implications, independent of any specific organization of responses. Here we define behavioural relevance as the significance of a cue to forthcoming behaviour, whether primarily motor or cognitive, and by non-motor resource implications we mean the need for sustained selective attention or working memory following behaviourally relevant cues that make a response likely. Selective attention is the mechanism by which subjects bias perceptual and cognitive processing in favour of objects and events that are relevant to their behavioural goals. Indeed, the BG and the frontal cortical areas with which they connect are considered to be of crucial importance in determining and acting upon the behavioural relevance of environmental stimuli according to environmental and internal contingencies (Saint-Cyr, 2003). Through this action the BG and related cortical systems are thought to ensure that the current cognitive focus is interrupted and available resources reallocated so that salient events can be processed with priority (Redgrave *et al.*, 1999a, b). Failure in this processing might contribute to the behavioural impairment following BG lesions (Levy & Dubois, 2006; Dujardin *et al.*, 2007) and functional neurosurgical procedures on the STN in particular (Drapier *et al.*, 2006, 2008).

Here we investigate whether the STN area may be involved in the management of any non-motor resource implications of environmental cues (over and above any role in the prospective preparation and programming of movement) by recording from the STN area in patients with Parkinson’s disease (PD) who had undergone implantation of this nucleus as a prelude to therapeutic high-frequency deep-brain stimulation (DBS). Patients were recorded as they performed a paradigm that separated warning cue presentation from processing related to the task to be completed. In addition, we evaluated whether activity evoked by the warning cue varied according to whether the eventual task to be performed was motor or cognitive and whether patients were receiving or withdrawn from dopaminergic therapy. In this way we provide evidence that the STN region is involved in the prospective management of non-motor resources, such as selective attention and working memory, following external stimuli that may require future responses, whether primarily motor or cognitive.

## Materials and methods

### Patients and surgery

Fourteen consecutive patients with idiopathic PD [two females, age 56 ± (SD) 6 years, disease duration 12 ± 5 years] who underwent implantation of DBS electrodes in the STN participated in the study. With the exception of patient 12, all patients were implanted bilaterally. Their clinical details are summarized in Table 1. All patients gave their informed written consent to take part in the study, which was approved by the local ethics committees of the different surgical centres according to the Declaration of Helsinki. The DBS electrode used was model 3389 (Medtronic Neurological Division, Minneapolis, MN, USA) with four platinum–iridium cylindrical surfaces (1.27 mm diameter and 1.5 mm length) and a contact-to-contact separation of 0.5 mm, except in Oxford where the 3387 model was used. Contact 0 was the most caudal and contact 3 was the most rostral. Electrode implantation was performed according to the standard procedures of each surgical centre. The intended coordinates for STN were 12 mm lateral from the midline, 3 mm behind the midcommissural point, and about 4 mm below the AC–PC line. Adjustments to the intended surgical coordinates were made according to the direct visualization of STN on individual pre-operative stereotactic T2-weighted magnetic resonance imaging, intra-operative stimulation and, in cases 1, 3, 5, 8–11 and 13, intra-operative microelectrode recordings. Post-operative CT or MRI imaging was performed in all patients to confirm targeting and suggested that at least one contact was within the STN. Five patients were implanted at the University Hospital Gasthuisberg, Leuven, Belgium; four patients were implanted at the Oxford Radcliffe Hospital, Oxford, UK; three patients were implanted at King’s College Hospital, London, UK; and two patients were implanted at the National Hospital for Neurology and Neurosurgery, London, UK. With one exception, none of the patients had any surgical complication, postoperative confusion or severe motor impairment that precluded understanding or performance of the test. The exception was case 13 in whom severe rest tremor precluded recording Off medication. None of the patients was cognitively impaired (Table 1).

**Table 1:** Clinical details

Case and surgical centre	Age (years and sex)	Handedness/most affected side	Disease duration (years)	Predominant symptoms	UPDRS part III On/Off drugs (max 108)	Medication, daily dose (mg)	MMSE, DRS-2 or WAIS-III
1 Leuven	49 M	R/R	10	On–Off fluctuations, dyskinesias	9/17	Levodopa 800 Pramipexole 3.15 Entacapone 800 Rasagiline 1	30 MMSE
2 Oxford	60 F	R/R	13	Severe Off rigidity, dystonia, freezing	11/33	Levodopa 800 Entacapone 800 Cabergoline 4	140 DRS-2
3 Leuven	66 M	R/L	17	On–Off fluctuations, dyskinesias	23/57	Levodopa 250 Entacapone 1000 Pergolide 3 Selegiline 10	27 MMSE
4 Oxford	64 M	R/R	8	On–Off fluctuations, dyskinesias, tremor	–	Levodopa 1200	109 DRS-2
5 London 1	57 M	R/R	22	Dyskinesias	31/50	Levodopa 50 Cabergoline 1 Apomorphine 4.5 mg/h plus 6 mg bolus	115 VIQ/94 PIQ
6 London 2	51 M	R/L	8	On–Off fluctuations, freezing	18/40	Levodopa 550	102 VIQ/121 PIQ
7 London 2	60 M	L/L	15	Dyskinesias, freezing	5/34	Levodopa 1000 Ropinirole 8	125 VIQ/155 PIQ
8 London 1	54 M	R/L	12	Tremor	18/37	Levodopa 1400	91 VIQ/98 PIQ
9 Leuven	56 F	R/R	13	On–Off fluctuations, dyskinesias	5/41	Levodopa 400 Pramipexole 2.8	28 MMSE
10 Leuven	53 M	R/L	11	On–Off fluctuations, dyskinesias	4/28	Levodopa 700 Entacapone 1400 Ropinirole 35	28 MMSE
11 Leuven	52 M	R/L	17	On–Off fluctuations, dyskinesias	10/37	Levodopa 200 Pramipexole 3.15 Amantadine 100 Selegiline 10	30 MMSE
12 Oxford	59 M	L/L	4	Tremor, rigidity	9/45	Levodopa 600 Ropinirole 24	132 DRS-2
13 London 1	46 M	L/R	8	Tremor	19/53	Levodopa 1000 Entacapone 1400 Rotigotine 16 Buspirone 15	122 VIQ/114 PIQ
14 Oxford	50 M	R/L	13	Dyskinesias, tremor	23/50	Apomorphine 4.5 mg/h	109 DRS-2

MMSE, Mini-Mental State Examination, max 30; DRS-2: Dementia Rating Scale-2, max 144; WAIS-III, Wechsler Adult Intelligence Scale-III (VIQ: Verbal IQ subscores; PIQ: Performance IQ subscores); London 1, King’s College Hospital; London 2, National Hospital for Neurology and Neurosurgery.

### Paradigm

**Fig. 1:**
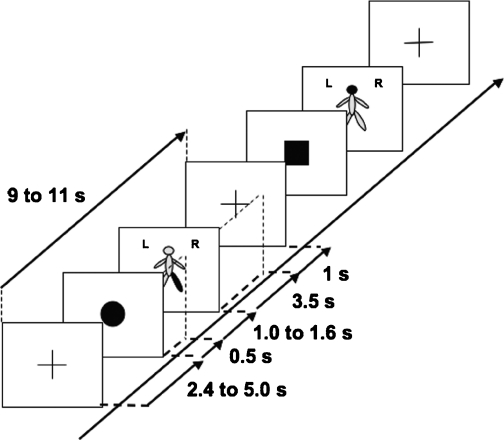
Schematic of two trials during the standard motor task. In this block type, patients were asked to copy the movement indicated by the body schema only when preceded by a circle. In the task, the body part to move was indicated in red. A similar sequence was used in the counting task.

Subjects were seated comfortably in a chair. The test consisted of three conditions: a ‘standard motor’ task and a ‘control motor’ task consisted of performing body movements according to visual cues presented on a laptop computer, while the remaining condition was a ‘counting’ task consisting of counting in silence identical cues while at rest. Each trial began with presentation of a warning cue. This could be a black circle (50% probability), square (25% probability) or triangle (25% probability), which substituted the fixation cross provided between trials to limit confounding eye movements (Fig. 1). The warning cue was followed by a schematic of the body with a segment highlighted in red (head, trunk, left arm, right arm, left leg or right leg, with equal probabilities). In the standard motor task, patients were instructed to move the highlighted body segment whenever preceded by a circle (target cue), but to ignore the body schema cue when this was preceded by either a non-target square or triangle (Fig. 1). This was reversed in the control motor task, in which patients were instructed to move the highlighted body segment whenever preceded by a square or triangle (target cue), but to ignore the body schema cue when this was preceded by a non-target circle. In the counting task, the patients were asked to remain at rest and to count silently in their mind how many left arms were highlighted in red after circles (target cues), ignoring non-target squares and triangles. At the end of the task the subjects were asked to report this number. The experiment began with some demonstration trials in which the intended movements were shown to the patient. These movements consisted of single and brief flexion of the neck, flexion of the trunk, left or right wrist extension, and left or right ankle dorsiflexion.

Each of the three tasks was repeated twice, the six blocks being presented in a pseudo-randomized order across patients. The task requirements were verbally explained to the subject prior to the start of each block. Blocks of a given task lasted 4 min, and were followed by 1–2 min rest. Each block of a given task consisted of 24 trials, giving a total number of 48 trials per condition. Each trial started with the presentation of a fixation cross at the centre of a portable PC screen, followed 2.4–5.0 s later by the warning cue of fixed duration (0.5 s) and by a 3.5-s duration second cue of a body schema presented at the centre of the screen. This was followed by a blank screen for 1 s, before the next trial began with reappearance of the fixation cross. Within each block, trials occurred pseudo-randomly, every 9–11 s. The two consecutive cues were presented with a randomized inter-cue duration of 1.0–1.6 s and were pseudo-randomized in type, thereby limiting prediction of the timing of the imperative cues and anticipation of the desired response. Moreover, in choosing similar overall probabilities of target and non-target cues we biased the experimental design so that evoked activity related to behavioural relevance would be stressed over that related to novelty. Thus, our experimental design differed from previous P300 and contingent negativity variation (CNV) paradigms in so far as the response to the warning cue was disambiguated from specific task-related motor or cognitive processing and behavioural relevance emphasized over novelty.

### Recordings

Patients were studied 5 ± 1 days post-operatively, in the interval between DBS electrode implantation and subsequent connection to a subcutaneous stimulator. Nine patients were first assessed after overnight withdrawal of antiparkinsonian medication, then 73 ± 18 min after a supra-maximal dose of levodopa (usual morning dose or 200 mg equivalent-levodopa, whichever the highest). Two further patients were first recorded On medication and then Off medication on two consecutive days. Due to time constraints, one patient was only assessed Off medication and two patients were only assessed On medication.

LFPs and analogue signals related to the cues were recorded through a Biopotential Analyser Diana (Sechenov Institute of Evolutionary Physiology and Biochemistry, St Petersburg, Russia). Deep brain activity was recorded monopolarly from the four contacts of each DBS electrode, referenced to linked earlobes, amplified, filtered (0.5–300 Hz) and sampled at a common rate of 1200 Hz. Signals were monitored online using software written in our laboratory. During recordings, we marked the timing of errors by patients related to the initial warning cue (either movement when not appropriate or no movement when the patient should have moved) and errors related to the second cue (movement of either wrong side or wrong body part).

### Analysis and statistics

**Fig. 2:**
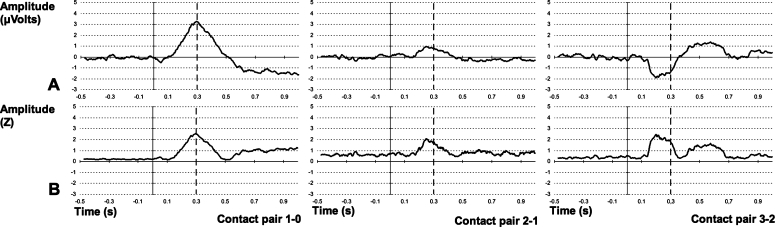
Averaged LFP activity from right STN in patient 1 during the standard motor task. Signals were averaged around the warning circle cue (thin continuous vertical line at time=0 s), and are shown before (A) and after (B) z-transformation and conversion to absolute values. The vertical dashed line indicates a latency of 300 ms after the cue. Averages have been smoothed using a moving average filter with a period of 30 data points.

Signals were exported for off-line processing in Spike 2 V6 software (Cambridge Electronic Design, UK). After reconstruction of a bipolar montage obtained by subtraction of the signal from adjacent electrode contacts, we removed any DC offset by filtering with a time constant of 1 s. The bipolar signal was then divided into trials from 0.5 s before to 1.0 s after each warning (circle/square/triangle) cue and averaged. The resulting data were exported to Excel software (Microsoft) for subsequent analysis. First, by way of normalization, the averaged signal (Fig. 2A) was z-transformed with respect to the period from 0.5 s before to 1 s after the warning cue. As bipolar signals can undergo polarity reversal across contact pairs these were then rectified by taking the absolute value of the normalized value to enable subsequent averaging across bipolar contacts (Fig. 2B). In analysing differences between conditions we opted for the conservative approach of using all bipolar contacts so as not to make any *a priori* assumptions about the localization of potential LFP generators and to avoid biases introduced by empirical selection of ‘best’ contact pairs.

Data from left and right subthalamic electrodes were assumed to be independent, affording up to 27 sides for analysis. Differences between signals from the 27 sides were explored using time-evolving serial two-tailed *t*-tests. In order to limit false-positive differences due to multiple comparisons, only those results where at least three consecutive *t*-tests were significant at *P*<0.05 were plotted. Additional statistical analyses including repeated-measures anovas were conducted using spss V12 (SPSS, Chicago, IL, USA). Here, significance was taken as *P*<0.05 and a trend towards an effect taken as *P*<0.1. Trends are mentioned in the Results, but are not considered further in the interpretation given in the Discussion, because of the risk of Type I error. Normal distribution of the data was confirmed using one-sample Kolmogorov–Smirnov tests.

## Results

The major analysis involved the comparison of the evoked potentials in the standard motor task with those in the counting task performed On and Off dopaminergic medication. These tasks were exactly matched in their mix of cue types. In a subsidiary analysis we compared the evoked potentials in the standard motor task with those in the control motor task performed On and Off dopaminergic medication. These two tasks reversed cues so that the circle was the target cue in the motor task, and the square or triangle were the target cues in the control motor task. This enabled us to show that the behavioural relevance of cues, rather than cue probability or form, was the critical variable.

### Behavioural data

Patients performed the tasks well. There were two types of error in the ‘standard moving task’. The first related to the warning cue (circle/square/triangle) and involved patients subsequently moving when they should not have or failing to move when they should have. Out of a total of 576 trials (24 blocks standard motor task) Off medication and 624 trials (26 blocks counting task) On medication there were 10 such errors Off medication (range 1–2, eight affected patients) and 11 errors On medication (range 1–3, six patients). The second type of error was related to the second (body schema) cue, and involved patients moving the wrong side or body segment. There were six such errors Off medication (range 1–2, five patients) and seven On medication (range 1–2, six patients). As we were primarily interested in the evoked response to the warning cue we removed those trials with the first type of error as attention to the warning cue could not be ensured in these instances. Thus, we analysed the signal related to 566/576 and 613/624 warning cues Off and On medication, respectively. In the counting task, Off medication, six patients counted wrongly in one block, but correctly in the remaining block. On medication, two patients counted wrongly in one block. Thus, there was a trend for more counting errors Off medication (24 blocks Off, 26 blocks On, Fisher’s exact test, *P*=0.1).

### Analysis of the signal in relation to the type of warning cue

**Fig. 3:**
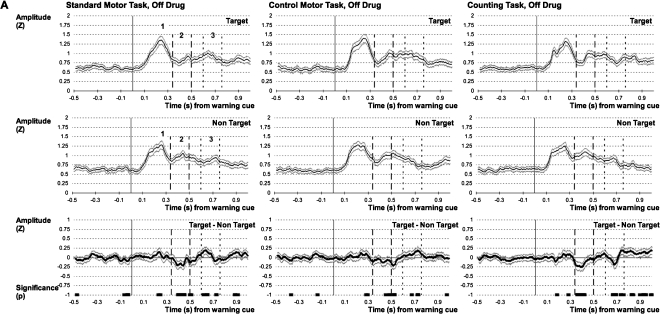

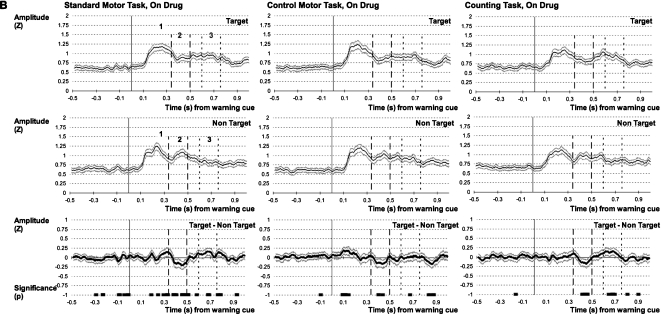
Grand averaged evoked potentials to target and non-target warning cues in the standard motor task (left column), control motor task (middle column) and counting (right column) task. (A) Off medication. The first panel represents the signals (mean ± SEM) evoked by target, the second panel represents the signal evoked by non-target, and the third panel represents the trace resulting from the subtraction of the two signals and the significant differences (*P*<0.05) between the signals evoked by the target and non-target cues sustained over=3 consecutive data points. (B) As above for On medication. Warning cue onset=0 s. Thick and thin dashed vertical lines represent the limits of activity periods 2 and 3, [340–500] and [600–760] ms after the warning cue onset, respectively. Grand averages have been smoothed using a moving average filter with a period of 30 data points.

**Fig. 4:**
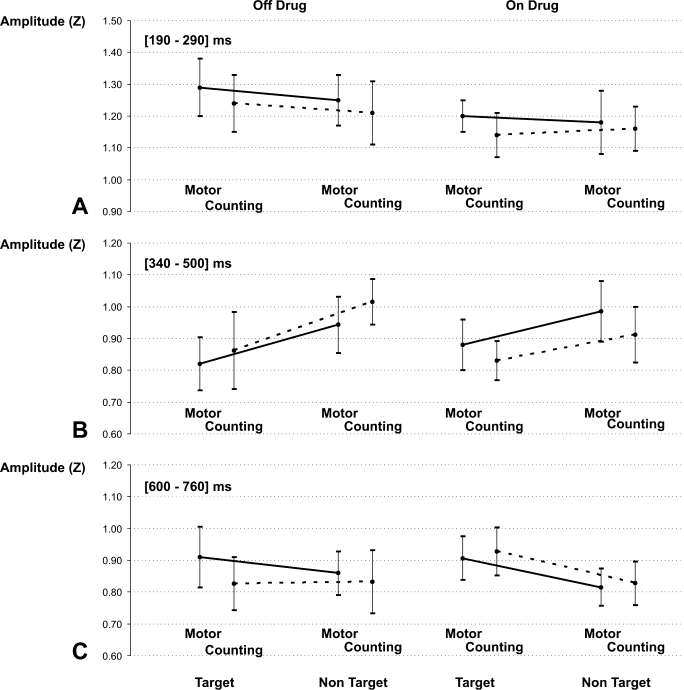
Mean amplitudes and standard deviation of the first (A), second (B) and third (C) activity periods Off (left column) and On (right column) medication for the target (circle, shown first) and non-target (square/triangle, shown second) warning cues in the standard moving and counting tasks. The solid line represents the difference in amplitude between ‘circle’ and ‘square/triangle’ in the standard motor task. The dashed line represents the same difference in amplitude for the counting task.

Twenty-three STN were recorded Off medication and 25 STN On medication in 14 patients. Averaged evoked responses to target and non-target cues in the standard motor and the counting tasks Off and On medication are illustrated in the left- and right-hand panels in Fig. 3. There was an initial peak at about 240 ms that was relatively invariant across cues, tasks and drug conditions, that was followed by later evoked activity characterized by its dependence on cue type and further modulated by medication, but relatively little by task. Time-evolving *t*-tests (Fig. 3) supported the above description, although they also suggested that the activity of longer latency might consist of two particular periods differentially affected by cue type (340–500 ms and 600–760 ms, as discussed below). We therefore analysed three time periods of interest. These are identified by the numbers 1, 2 and 3 in Fig. 3, and their mean amplitudes are summarized in Fig. 4. There was no asymmetry of any of the components between left and right sides (data not shown).

Evoked activity during the first time period consisted of a discrete peak. Its peak latency was 240 ± 35 ms, and it was of similar shape and amplitude irrespective of whether warning cues were target (circle) or non-target (square/triangle), the task was the standard motor one or counting, or patients were Off or On medication. The LFP amplitude over the first time period was taken as the average amplitude over a period of 100 ms centred on the peak (190–290 ms). A repeated-measures anova using factor ‘medication’ (Off and On) × ‘task (standard motor and counting) × ‘cue’ (target and non-target) showed no effect for the factor ‘cue’ (*F*_1,62_ = 2.026, *P*=0.160), and no interaction between ‘medication’ × ‘task’ (*F*_1,62_=0.080, *P*=0.778), ‘cue’ × ‘task’ (*F*_1,62_=0.569, *P*=0.453) or ‘medication’ × ‘task’ × ‘cue’ (*F*_1,62_=2.079, *P*=0.154). There were trends for the factors ‘medication’ (*F*_1,62_=3.415, *P*=0.069) and ‘task’ (*F*_1,62_=3.185, *P*=0.079), and for the interaction between ‘medication’ × ‘cue’ (*F*_1,62_=3.172, *P*=0.080). In line with the above, time-evolving *t*-tests did not reveal any consistent difference in the amplitude of the signals with relation to the type of warning cue in the four conditions (in the left- and right-hand panels in Fig. 3).

Evoked activity during the second time period was characterized by its dependence on whether the warning cue was target (circle) or non-target (square/triangle) in nature, regardless of whether the patient was engaged in the standard motor or counting task, and regardless of treatment state. A discrete peak could not be consistently identified, but time-evolving *t*-tests identified a period from between 340 and 500 ms over which the LFP evoked by the target was of lower amplitude than that evoked by the non-target cue (in the left- and right-hand panels in Fig. 3). This time period was further analysed using an anova structured as above. This showed a significant effect for the factor ‘cue’ (*F*_1,62_=8.154, *P*=0.006) and a significant interaction between ‘medication’ × ‘task’ (*F*_1,62_=9.277, *P*=0.003), no effect for the factor ‘medication’ (*F*_1,62_=0.488, *P*=0.488) or ‘task’ (*F*_1,62_=0.344, *P*=0.560), and no interaction between ‘medication’ × ‘cue’ (*F*_1,62_=0.519, *P*=0.474), ‘cue’ × ‘task’ (*F*_1,62_= 0.163, *P*=0.687) or ‘medication’ × ‘task’ × ‘cue’ (*F*_1,62_=1.458, *P*=0.232). Figure 4 summarizes the data from the 340–500 ms period, and indicates that the activity elicited by both the target circle and non-target square/triangle cues was greater in the counting task Off compared with On dopaminergic therapy.

Evoked activity during the third time period was also characterized by its dependence on whether the warning cue was target or non-target in nature. A discrete peak could not be consistently identified, but time-evolving *t*-tests identified a period from between 600 and 760 ms over which the LFP evoked by target cues was of higher amplitude than that evoked by non-target cues, except when the counting task was performed OFF treatment (Figs 3 and 4). An anova using factor ‘medication’ (Off and On) × ‘task’ (standard motor and counting) × ‘cue’ (target and non-target) showed a significant effect for the factor ‘cue’ (*F*_1,62_=4.853, *P*=0.031) and a significant interaction between ‘medication’ × ‘cue’ (*F*_1,62_=7.836, *P*=0.007), no effect for the factors ‘medication’ (*F*_1,62_=0.138, *P*=0.712) or ‘task’ (*F*_1,62_=1.137, *P*=0.290), and no interaction between ‘cue’ × ‘task’ (*F*_1,62_=1.664, *P*=0.202) or ‘medication’ × ‘task’ × ‘cue’ (*F*_1,62_=0.445, *P*=0.507; Fig. 4). There was a trend for an interaction between ‘medication’ × ‘task’ (*F*_1,62_=2.993, *P*=0.089). The ‘medication’ × ‘cue’ interaction arose because the difference between the evoked activity to target and non-target cues in each task was greater On medication than Off medication. Thus, the behavioural relevance of cues differentially modulated evoked activity in the second and third time periods of interest, respectively (with the only exception being the effect on evoked activity during the third period in the counting task OFF therapy).

### Independency from the intrinsic characteristics of the target cue

The above suggests that the second and third periods of evoked activity may be modulated by the behavioural relevance of the cue. However, the difference in the response to the target and non-target cues might also have arisen from the difference in cue morphology and probability (target circle 50% of trials vs. non-target square/triangle, each with 25% of trials). Accordingly, we controlled for this by including a control motor task in which the behavioural relevance of the cues was reversed so that we presented target square and triangle cues, each comprising 25% of all trials, and non-target circles comprising 50% of all trials. The evoked activity in these trials in which the square and triangle instructed forthcoming movement was then compared with that in the standard motor task in which the circle was the target cue instructing forthcoming movement. The grand average evoked potentials were similar (middle panels in Fig. 3), so the same three periods were chosen for analysis as above. Here, a persistent main effect for cue (target and non-target, regardless of cue form) and lack of an effect of task (motor and control motor), including any interaction with cue would reinforce the interpretation that differences in late evoked potentials were due to the behavioural relevance or otherwise of the triggering cues.

### First time period

A repeated-measures anova using factor ‘medication’ (Off and On) × ‘task’ (standard motor and control motor) × ‘cue’ (target and non-target) showed no effect for the factor ‘task’ (*F*_1,62_=0.021, *P*=0.885], and no interaction between ‘medication’ × ‘task’ (*F*_1,62_=0.082, *P*=0.775), ‘medication’ × ‘cue’ (*F*_1,62_=2.719, *P*=0.104), ‘cue’ × ‘task’ (*F*_1,62_=0.225, *P*=0.637) or ‘medication’ × ‘task’ × ‘cue’ (*F*_1,62_=0.214, *P*=0.646). There was a trend for an effect for the factors ‘medication’ (*F*_1,62_=2.870, *P*=0.095) and ‘cue’ (*F*_1,62_=3.766, *P*=0.057). The trend in favour of an effect of medication matched a similar trend in the standard paradigm and might suggest a weak effect of treatment status on evoked activity with the earliest latency.

### Second time period

A similar anova showed a significant effect for the factor ‘cue’ (*F*_1,62_=12.087, *P*=0.001), no effect for the factors ‘medication’ (*F*_1,62_=1.434, *P*=0.236), ‘task’ (*F*_1,62_=0.478, *P*=0.492), and no interaction between ‘medication’ × ‘task’ (*F*_1,62_=1.049, *P*=0.31), ‘medication’ × ‘cue’ (*F*_1,62_=0.249, *P*=0.619), ‘cue’ × ‘task’ (*F*_1,62_=0.206, *P*=0.651) or ‘medication’ × ‘task’ × ‘cue’ (*F*_1,62_=0.002, *P*=0.969). There was therefore no effect of cue form, although the behavioural relevance of cues remained important.

### Third time period

A similar anova showed a significant effect for the factor ‘cue’ (*F*_1,62_=11.472, *P*=0.001) and a significant interaction between ‘medication’ × ‘task’ × ‘cue’ (*F*_1,62_=5.981, *P*=0.017), no effect for the factors ‘medication’ (*F*_1,62_=0.066, *P*=0.798), ‘task’ (*F*_1,62_=0.594, *P*=0.444), and no interaction between ‘medication’ × ‘task’ (*F*_1,62_=1.182, *P*=0.281), ‘medication’ × ‘cue’ (*F*_1,62_=0.059, *P*=0.809), ‘cue’ × ‘task’ (*F*_1,62_=0.474, *P*=0.494). The interaction between ‘medication’ × ‘task’ × ‘cue’ was further analysed using *t*-tests, and was found to be due to a difference between target and non-target cues that was greater Off medication in the control motor task (*P*=0.008) but not standard motor task (*P*=0.173). Nevertheless, the behavioural relevance of cues remained important, as indexed by the main effect for cue.

### Origin of LFP activity

**Fig. 5:**
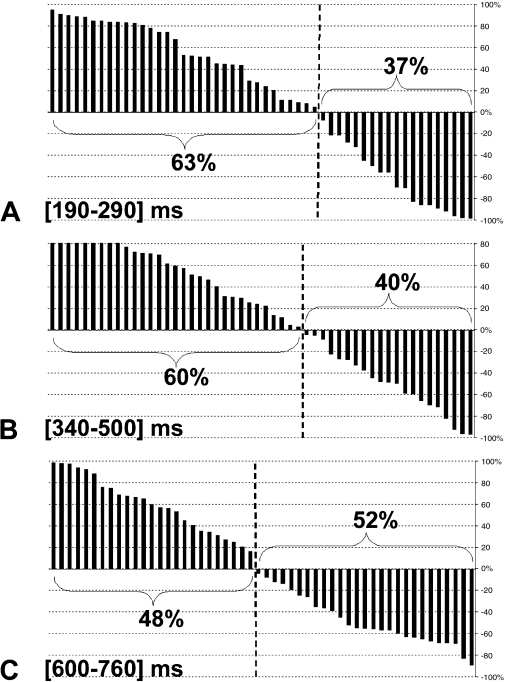
Distribution of the LFP amplitude during the first (A), second (B) and third (C) activity periods normalized to the contact pair with the maximum amplitude from each electrode (in the standard motor task). Negative values indicate polarity inversion relative to the sign of the contact pair with the maximum amplitude (defined as +100%).

We also determined whether LFPs reflected activities in local generators or volume conduction from distant, possibly cortical, sources. To address this point, the bipolar signals from each contact pair in the standard motor task were z-transformed with respect to the period from 0.5 s before to 1 s after the warning cue. The amplitude of the signal from each contact pair was then averaged over each of the three time periods. In each time period, the maximum amplitude from the three contact pairs from each electrode was defined as +100% and the amplitude of the two remaining pairs normalized to that maximum. The contact pairs with the maximum amplitude for each electrode were arbitrarily distributed (32%, 32% and 36% at contact 01, 12 and 23, respectively, for the first time period; 23%, 38%, 38% for the second time period and 50%, 27%, 23% for the third period). The relative amplitudes of the two remaining contact pairs from each electrode were then plotted in descending order of normalized amplitude (Fig. 5). The distribution of relative amplitudes was similar for each period of activity. Signals dropped in amplitude, and even reversed in polarity in 37%, 40% and 52% of instances for the first, second and third activity periods, respectively, consistent with local generators (Rektor *et al.*, 2004, 2005).

## Discussion

The aim of this study was to determine whether the STN area is involved in the evaluation and management of the behavioural relevance of environmental cues and any non-motor resource implications, such as the need for sustained selective attention or working memory following salient cues. The critical aspect of our paradigm was the dissociation of the response to the warning cue from any programming related to execution of the specific task, whether primarily motor or cognitive, as the nature of this was only cued later and after a variable delay. Our experimental design therefore differed from previous P300 and CNV paradigms in so far as the response to the warning cue was largely disambiguated from expectancy and prediction related to the timing of the imperative cue and from specific task-related motor or cognitive processing such as selection, planning and execution of required behaviour. Likewise, the warning cue studied here should not be confused with standard externally triggered movement paradigms (Jahanshahi *et al.*, 1995) that have more in common with the imperative cue delivered in our paradigm.

We found that warning cues evoked a complex temporal sequence of activities in the subthalamic area that differed in their pattern according to the behavioural relevance of cues and the dopaminergic state. On the other hand, the pattern of evoked activities was similar in both motor and cognitive tasks, in keeping with the independence of our warning cues from the nature of the final response and in line with our hypothesis that evoked activities partly reflect the deployment of non-motor resources, such as sustained attention or working memory following salient cues. Before expanding on the potential relevance of our findings we should, however, consider several important limitations common to the experimental approach taken here.

## Experimental limitations

It is important to stress that we did not consider the ‘non-target’ cues to be devoid of behavioural relevance, which is not an all-or-nothing attribute. For example, it could be posited that the non-target warning cue actually elicited an inhibition of activity, as the STN has often been implicated in the inhibition of motor responses (Cooper *et al.*, 1994; Baunez & Robbins, 1997; Phillips & Brown, 2000; Kuhn *et al.*, 2004; Aron & Poldrack, 2006). However, our paradigm meant that there was no clear motor or cognitive response to be inhibited following presentation of non-target warning cues. Nevertheless, non-target cues could have signalled that attentional and working memory resources could be spared. Thus, the critical difference between our target and non-target cues is not that one was informative and one was not, but that there was a difference in the behavioural relevance of each. Thus, any effect of cue on the pattern of evoked activity implies that the behavioural relevance of the cue did modulate post-cue processing in the subthalamic region.

Recordings were necessarily made in patients with PD, and their execution was inevitably affected by time constraints and their interpretation influenced by what can be said about the physiological and anatomical basis of examined phenomena in clinical studies. First of all, On and Off medication states were not counterbalanced in terms of their order of presentation. Thus, the majority of patients were recorded in the Off and On medication states on the same day, with the Off state being recorded first. Accordingly, differences between drug states may have been confounded by learning and fatigue effects over time, although the results in the two patients recorded On medication first followed a similar pattern to those recorded in the remaining patients (see Fig. S1). Second, in the foregoing discussion we will tend to frame our findings in the context of essentially physiological rather than pathological processes, even though we have no direct evidence to refute the possibility that evoked LFP activities are related to the parkinsonian state. On the other hand, the reports of P300-like responses in the BG of patients with epilepsy would argue that BG involvement in the orientation of attention may be a generic physiological function that is irrespective of disease process (Rektor *et al.*, 2004, 2005). In addition, the presence of, or increase in, LFP activities in patients with PD following treatment with levodopa provides further weak evidence that these features may be essentially physiological. It is also important to consider what the implied dependence on dopaminergic state entails, for it is likely that treatment with levodopa is primarily promoting the tonic rather than the phasic actions of dopamine. Finally, it is important to recall the limitations surrounding the precise localization of the different electrode contacts. These have been previously discussed at length (Cassidy *et al.*, 2002; Paradiso *et al.*, 2003; Williams *et al.*, 2003; Kuhn *et al.*, 2004; Rektor *et al.*, 2004) and it is perhaps best to denote the contacts as lying in and picking up from the subthalamic region rather than the STN itself (Kuhn *et al.*, 2004; Balaz *et al.*, 2008). This is particularly relevant in the current study as we averaged activity from all electrode contact pairs.

Finally, although we identified changes in the pattern of evoked activities according to the behavioural significance of the warning cue that we related, in part, to the deployment of non-motor resources, such as sustained selective attention or working memory following salient cues, we did not demonstrate that such anticipatory resourcing lead to a functional advantage in our paradigm. This was because the nature of our paradigm, in which final motor responses could involve any limb, the trunk or neck, together with the need to keep our set-up portable (patients were necessarily studied in several surgical centres), prohibited electromyographic recordings of response times.

### Interpretation of the results in relation to action programming

Despite the above considerations several important observations can be made. The results show an early evoked LFP component in the subthalamic area related to cue appearance that was relatively unaffected by its context (i.e. whether target or non-target or seen during the motor or counting tasks). This may contribute to the initial orientating of attention induced by a new stimulus. In this regard, it would be interesting to assess whether this evoked component occurs irrespective of the modality of the novel cue, increases with the rarity of the target cue and can occur without active scanning of the environment. In contrast, the early evoked activity was followed by activities over two later periods whose magnitudes were significantly influenced by the behavioural relevance of the cues. The nature of our paradigm excluded specific motor planning and programming prior to the appearance of the imperative cue, and thus the longer latency activity evoked by the warning cues may reflect non-motor processing such as that related to changes in attentional demands and recruitment of working memory (as the target must be both selected for its behavioural relevance and this information held in working memory so as to enable the appropriate response to the imperative cue). Accordingly, there was no difference in response to behaviourally relevant cues that then fashioned a motor or cognitive response to later events.

The later periods of activity were also characterized by their partial but significant modulation by levodopa treatment. Although the increased response to non-target cues over 340–500 ms was unaffected by levodopa, treatment with the later did decrease the magnitude of the evoked activity to both target and non-target warning cues in the cognitive task. In contrast, whether target cues elicited bigger evoked responses over 600–760 ms than non-target cues did depend on the drug treatment state in the motor task. This evidence of dopamine modulation of activities sensitive to the behavioural relevance of cues is consistent with other evidence that dopamine enhances the processing of salient environmental signals, thereby assisting appropriate responses (Nicola *et al.*, 2000). Note that we found no lateralization of evoked responses following the warning cues, implying that activity related to the evaluation of the novelty and saliency of external cues may be bilaterally represented, at least at the subthalamic level. Similar bilateral STN activities have been reported during movement preparation (Paradiso *et al.*, 2003; Fawcett *et al.*, 2007).

The activities evoked in the STN and their variable dependency on context bear some similarities with the mismatch negativity (MMN), P300a and P300b recorded under similar circumstances at the level of the cerebral cortex. The first subcortical component overlapped in latency with both the MMN and P300a and, like these, was not modulated by context. Both the MMN and P300a have been implicated in the orientating of attention following a novel stimulus (Posner & Petersen, 1990). In contrast, the two later components recorded in the STN region had latencies overlapping with the P300b elicited when novel stimuli are also task-relevant. The cortical P300a and some portion of P300b are affected by dopaminergic activity (Polich, 2007), but in the reverse direction to the effect of dopamine in the STN area found here. The CNV elicited by paired warning and imperative cues also bears some similarities to the activities evoked in the current paradigm, although the former conflates orientation of attention with expectancy and specific motor-related processing. The CNV also increases, rather than decreases, following dopaminergic treatment (Amabile *et al.*, 1986).

The similarities with some cortical evoked activities raise two possibilities. Either the subcortical activities recorded here might represent volume conduction from cortex or some portion of the subcortical-evoked activities might represent neural processes at the BG end of circuits between the BG and frontal cortex concerned with attentional processes leading up to behavioural responses (Kermadi & Boussaoud, 1995; Boussaoud & Kermadi, 1997; Rouiller *et al.*, 1998). Consideration of the pattern of evoked activity across electrode contact pairs, and particularly polarity reversal, argues against volume conduction. Further, the human striatum and globus pallidus exhibit both P300-like LFP and CNV-like LFP activities (Rektor *et al.*, 2004, 2005) and, importantly, corresponding changes at the single-unit level (Kropotov & Ponomarev, 1991). Within these nuclei there is a short-latency stimulus-bound response (lasting from 100 to about 500 ms) that is no different for relevant and irrelevant (non-target) stimuli and not affected by task. Then there is a longer latency response (300–800 ms) when cues are behaviourally relevant (Kropotov & Ponomarev, 1991), rather as in the present study. A P300-like potential with a latency of 260–295 ms has also been recently recorded in the STN following a modified auditory oddball task (Balaz *et al.*, 2008). However, unlike the present investigation, previous studies used paradigms that conflated contextual factors with response selection, so that from these alone it was difficult to divorce contextual influences from selection and processing of motor or cognitive responses (Kropotov & Ponomarev, 1991). Nevertheless, these data suggest that the evoked activity in the STN region may be propagated to globus pallidus and then to cortex via the ventrolateral thalamus, where P300-like activity can also be recorded (Kropotov & Ponomarev, 1991). This is not to say that potentials related to cue novelty and behavioural relevance arise in the STN, for the STN may be just one node in a circuit elaborating such responses. For example, it is important to note that functional magnetic resonance imaging studies have shown that striatal activation in the human reflects the degree of stimulus saliency (Zink *et al.*, 2006).

These observations suggest that the BG are intimately involved in the evaluation of changes in the environment and of their potential resource implications. This process is partly modulated by dopamine. Our findings are in accord with the view that dopaminergic input into the BG has a general role in the processing of salient events, irrespective of any specific function in coding reward value or prediction error (Redgrave *et al*., 1999a, b). They also serve to stress that BG function is not limited to the processing of internally guided actions (Mushiake & Strick, 1995; Jueptner & Weiller, 1998; van Donkelaar *et al.*, 2000).

## Abbreviations

BG: basal ganglia
CNV: contingent negativity variation
DBS: deep-brain stimulation
LFPs: local field potentials
MMN: mismatch negativity
PD: Parkinson’s disease
STN: subthalamic nucleus

## Supporting information

Fig. S1. Comparison of grand averaged evoked potentials to target and non-target warning cues in the standard motor and counting tasks according to the order of dopamine administration.

## References

[b1] Alegre M, Alonso-Frech F, Rodriguez-Oroz MC, Guridi J, Zamarbide I, Valencia M, Manrique M, Obeso JA, Artieda J (2005). Movement-related changes in oscillatory activity in the human subthalamic nucleus: ipsilateral vs. contralateral movements. Eur. J. Neurosci..

[b2] Alonso-Frech F, Zamarbide I, Alegre M, Rodriguez-Oroz MC, Guridi J, Manrique M, Valencia M, Artieda J, Obeso JA (2006). Slow oscillatory activity and levodopa-induced dyskinesias in Parkinson’s disease. Brain.

[b3] Amabile G, Fattapposta F, Pozzessere G, Albani G, Sanarelli L, Rizzo PA, Morocutti C (1986). Parkinson disease: electrophysiological (CNV) analysis related to pharmacological treatment. Electroencephalogr. Clin. Neurophysiol..

[b4] Androulidakis AG, Kuhn AA, Chen CC, Blomstedt P, Kempf F, Kupsch A, Schneider GH, Doyle L, Dowsey-Limousin P, Hariz MI, Brown P (2007). Dopaminergic therapy promotes lateralized motor activity in the subthalamic area in Parkinson’s disease. Brain.

[b5] Aron AR, Poldrack RA (2006). Cortical and subcortical contributions to Stop signal response inhibition: role of the subthalamic nucleus. J. Neurosci..

[b6] Balaz M, Rektor I, Pulkrabek J (2008). Participation of the subthalamic nucleus in executive functions: an intracerebral recording study. Mov. Disord..

[b7] Baunez C, Robbins TW (1997). Bilateral lesions of the subthalamic nucleus induce multiple deficits in an attentional task in rats. Eur. J. Neurosci..

[b8] Baunez C, Humby T, Eagle DM, Ryan LJ, Dunnett SB, Robbins TW (2001). Effects of STN lesions on simple vs choice reaction time tasks in the rat: preserved motor readiness, but impaired response selection. Eur. J. Neurosci..

[b9] Boussaoud D, Kermadi I (1997). The primate striatum: neuronal activity in relation to spatial attention versus motor preparation. Eur. J. Neurosci..

[b10] Brucke C, Kupsch A, Schneider GH, Hariz MI, Nuttin B, Kopp U, Kempf F, Trottenberg T, Doyle L, Chen CC, Yarrow K, Brown P, Kuhn AA (2007). The subthalamic region is activated during valence-related emotional processing in patients with Parkinson’s disease. Eur. J. Neurosci..

[b11] Cassidy M, Mazzone P, Oliviero A, Insola A, Tonali P, Di Lazzaro V, Brown P (2002). Movement-related changes in synchronization in the human basal ganglia. Brain.

[b12] Cooper JA, Sagar HJ, Tidswell P, Jordan N (1994). Slowed central processing in simple and go/no-go reaction time tasks in Parkinson’s disease. Brain.

[b13] Devos D, Defebvre L (2006). Effect of deep brain stimulation and L-Dopa on electrocortical rhythms related to movement in Parkinson’s disease. Prog. Brain Res..

[b14] van Donkelaar P, Stein JF, Passingham RE, Miall RC (2000). Temporary inactivation in the primate motor thalamus during visually triggered and internally generated limb movements. J. Neurophysiol..

[b15] Doyle LM, Kuhn AA, Hariz M, Kupsch A, Schneider GH, Brown P (2005). Levodopa-induced modulation of subthalamic beta oscillations during self-paced movements in patients with Parkinson’s disease. Eur. J. Neurosci..

[b16] Drapier D, Drapier S, Sauleau P, Haegelen C, Raoul S, Biseul I, Peron J, Lallement F, Rivier I, Reymann JM, Edan G, Verin M, Millet B (2006). Does subthalamic nucleus stimulation induce apathy in Parkinson’s disease?. J. Neurol..

[b17] Drapier D, Peron J, Leray E, Sauleau P, Biseul I, Drapier S, Le Jeune F, Travers D, Bourguignon A, Haegelen C, Millet B, Verin M (2008). Emotion recognition impairment and apathy after subthalamic nucleus stimulation in Parkinson’s disease have separate neural substrates. Neuropsychologia.

[b18] Dujardin K, Sockeel P, Devos D, Delliaux M, Krystkowiak P, Destee A, Defebvre L (2007). Characteristics of apathy in Parkinson’s disease. Mov. Disord..

[b19] Fawcett AP, Cunic D, Hamani C, Hodaie M, Lozano AM, Chen R, Hutchison WD (2007). Saccade-related potentials recorded from human subthalamic nucleus. Clin. Neurophysiol..

[b20] Jahanshahi M, Jenkins IH, Brown RG, Marsden CD, Passingham RE, Brooks DJ (1995). Self-initiated versus externally triggered movements. I. An investigation using measurement of regional cerebral blood flow with PET and movement-related potentials in normal and Parkinson’s disease subjects. Brain.

[b21] Jueptner M, Weiller C (1998). A review of differences between basal ganglia and cerebellar control of movements as revealed by functional imaging studies. Brain.

[b22] Kermadi I, Boussaoud D (1995). Role of the primate striatum in attention and sensorimotor processes: comparison with premotor cortex. Neuroreport.

[b23] Kropotov JD, Ponomarev VA (1991). Subcortical neuronal correlates of component P300 in man. Electroencephalogr. Clin. Neurophysiol..

[b24] Kuhn AA, Williams D, Kupsch A, Limousin P, Hariz M, Schneider GH, Yarrow K, Brown P (2004). Event-related beta desynchronization in human subthalamic nucleus correlates with motor performance. Brain.

[b25] Levy R, Dubois B (2006). Apathy and the functional anatomy of the prefrontal cortex-basal ganglia circuits. Cereb. Cortex.

[b26] Middleton FA, Strick PL (2000). Basal ganglia output and cognition: evidence from anatomical, behavioral, and clinical studies. Brain Cogn..

[b27] Mushiake H, Strick PL (1995). Pallidal neuron activity during sequential arm movements. J. Neurophysiol..

[b28] Nicola SM, Surmeier J, Malenka RC (2000). Dopaminergic modulation of neuronal excitability in the striatum and nucleus accumbens. Annu. Rev. Neurosci..

[b29] Paradiso G, Saint-Cyr JA, Lozano AM, Lang AE, Chen R (2003). Involvement of the human subthalamic nucleus in movement preparation. Neurology.

[b30] Phillips JM, Brown VJ (2000). Anticipatory errors after unilateral lesions of the subthalamic nucleus in the rat: evidence for a failure of response inhibition. Behav. Neurosci..

[b31] Polich J (2007). Updating P300: an integrative theory of P3a and P3b. Clin. Neurophysiol..

[b32] Posner MI, Petersen SE (1990). The attention system of the human brain. Annu. Rev. Neurosci..

[b33] Priori A, Foffani G, Pesenti A, Bianchi A, Chiesa V, Baselli G, Caputo E, Tamma F, Rampini P, Egidi M, Locatelli M, Barbieri S, Scarlato G (2002). Movement-related modulation of neural activity in human basal ganglia and its L-DOPA dependency: recordings from deep brain stimulation electrodes in patients with Parkinson’s disease. Neurol. Sci..

[b34] Redgrave P, Prescott TJ, Gurney K (1999a). Is the short-latency dopamine response too short to signal reward error?. Trends Neurosci..

[b35] Redgrave P, Prescott TJ, Gurney K (1999b). The basal ganglia: a vertebrate solution to the selection problem?. Neuroscience.

[b36] Rektor I, Bares M, Kanovsky P, Brazdil M, Klajblova I, Streitova H, Rektorova I, Sochurkova D, Kubova D, Kuba R, Daniel P (2004). Cognitive potentials in the basal ganglia-frontocortical circuits. An intracerebral recording study. Exp. Brain Res..

[b37] Rektor I, Bares M, Brazdil M, Kanovsky P, Rektorova I, Sochurkova D, Kubova D, Kuba R, Daniel P (2005). Cognitive- and movement-related potentials recorded in the human basal ganglia. Mov. Disord..

[b38] Rouiller EM, Tanne J, Moret V, Kermadi I, Boussaoud D, Welker E (1998). Dual morphology and topography of the corticothalamic terminals originating from the primary, supplementary motor, and dorsal premotor cortical areas in macaque monkeys. J. Comp. Neurol..

[b39] Saint-Cyr JA (2003). Frontal-striatal circuit functions: context, sequence, and consequence. J. Int. Neuropsychol. Soc..

[b40] Temel Y, Blokland A, Steinbusch HW, Visser-Vandewalle V (2005). The functional role of the subthalamic nucleus in cognitive and limbic circuits. Prog. Neurobiol..

[b41] Williams D, Kuhn A, Kupsch A, Tijssen M, van Bruggen G, Speelman H, Hotton G, Yarrow K, Brown P (2003). Behavioural cues are associated with modulations of synchronous oscillations in the human subthalamic nucleus. Brain.

[b42] Williams D, Kuhn A, Kupsch A, Tijssen M, van Bruggen G, Speelman H, Hotton G, Loukas C, Brown P (2005). The relationship between oscillatory activity and motor reaction time in the parkinsonian subthalamic nucleus. Eur. J. Neurosci..

[b43] Zink CF, Pagnoni G, Chappelow J, Martin-Skurski M, Berns GS (2006). Human striatal activation reflects degree of stimulus saliency. Neuroimage.

