# Supplementary material for: Involvement of the subthalamic nucleus in engagement with behaviourally relevant stimuli
Source: Eur J Neurosci. 2009 Mar;29(5):931–42. doi: 10.1111/j.1460-9568.2009.06635.x (PMC2695155; doi:10.1111/j.1460-9568.2009.06635.x)
Supplement: Supplementary file 1 [file ejn0029-0931-SD1.doc]

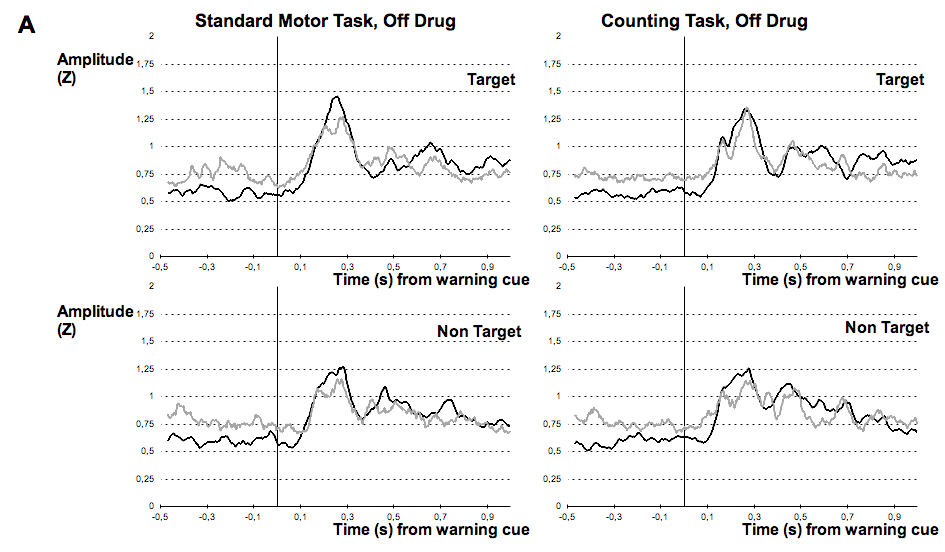


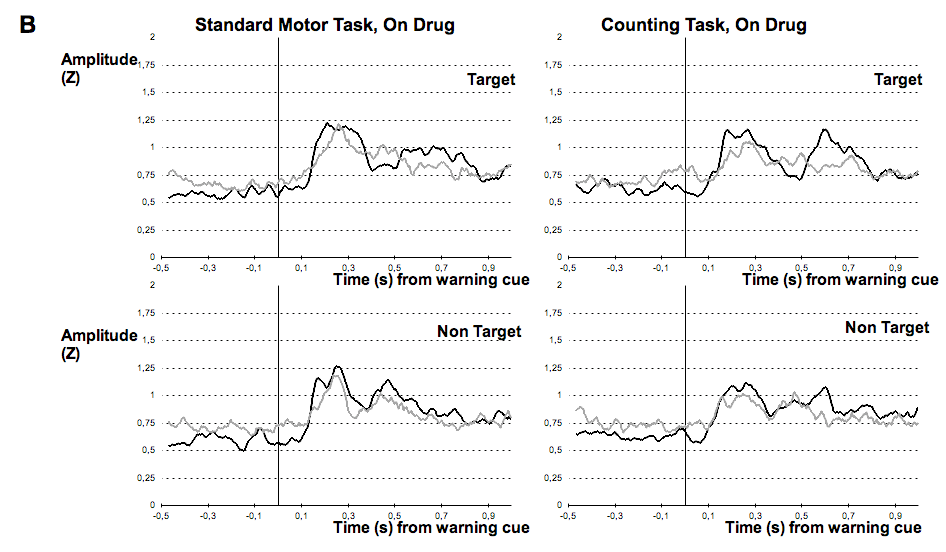


**Fig. S1**. Comparison of grand averaged evoked potentials to target and non-target warning cues in the standard motor and counting tasks according to the order of dopamine administration. The black line represents the average of the evoked potentials from the nine patients who were first recorded Off medication and then On medication and the one patient who was only assessed Off medication. The grey line represents the average from the two patients who were first recorded On medication and then Off medication and the two patients who were only assessed On medication. (A) Off medication. The first panel represents the signals evoked by target and the second panel represents the signal evoked by non-target warning cues. (B) As above for On medication. Warning cue onset = 0 s. Grand averages have been smoothed using a moving average filter with a period of 30 data points.
